# Prevalence and Characterization of Extended-Spectrum β-Lactamase-Producing *Escherichia coli* Isolated from Dogs and Cats in South Korea

**DOI:** 10.3390/antibiotics12040745

**Published:** 2023-04-13

**Authors:** Ji-Hyun Choi, Md. Sekendar Ali, Bo-Youn Moon, Hee-Young Kang, Su-Jeong Kim, Hyun-Ju Song, Abraham Fikru Mechesso, Dong-Chan Moon, Suk-Kyung Lim

**Affiliations:** 1 Bacterial Disease Division, Animal and Plant Quarantine Agency, 177 Hyeksin 8-ro, Gimcheon-si 39660, Republic of Korea; duddnjs2009@naver.com (J.-H.C.); alipharm@iiuc.ac.bd (M.S.A.); qiamby@korea.kr (B.-Y.M.); kanghy7734@korea.kr (H.-Y.K.); kimsujeong27@korea.kr (S.-J.K.); shj0211@korea.kr (H.-J.S.); 2 Department of Pathology and Microbiology, College of Medicine, University of Nebraska Medical Center, Omaha, NE 68198-5900, USA; afikru110@gmail.com; 3 Division of Antimicrobial Resistance Research, Center for Infectious Diseases Research, Korea Disease Control and Prevention Agency, Cheongju 28159, Republic of Korea

**Keywords:** β-lactamase, *bla*_CTX-M_, *bla*_CMY-2_, companion animals, *E. coli*, mutation, quinolone

## Abstract

Overall, 836 *Escherichia coli* isolates (695 isolates from dogs and 141 from cats) were recovered from the diarrhea, skin/ear, urine, and genitals of dogs and cats between 2018 and 2019. Cefovecin and enrofloxacin resistance were noted in 17.1% and 21.2% of *E. coli* isolates, respectively. The cefovecin and enrofloxacin resistance rates were higher in dog isolates (18.1% and 22.9%) compared with the rates in cat isolates (12.1%, 12.8%). Interestingly, resistance to both antimicrobials was noted in 10.8% (90/836) of the isolates, predominantly in isolates from dogs. *bla*_CTX-M-14_, *bla*_CTX-M-15_, and *bla*_CMY-2_ were the most frequent extended-spectrum β-lactamase/plasmid-mediated AmpC β-lactamase (ESBL/AmpC)- gene types. The co-existence of *bla*_CTX-M and_ *bla*_CMY-2_ was noted in six *E. coli* isolates from dogs. Sequencing analysis demonstrated that S83L and D87N in *gyrA* and S80I in *parC* were the most frequent point mutations in the quinolone resistance-determining regions of the cefovecin and enrofloxacin-resistant isolates. A total of 11 isolates from dogs carried the plasmid-mediated quinolone resistance genes (six *aac(6’)-Ib-cr*, four *qnrS*, and one *qnrB*), while only two cat isolates carried the *qnrS* gene. Multilocus sequence typing of the cefovecin and enrofloxacin-resistant isolates revealed that sequence type (ST)131 *E. coli* carrying *bla*_CTX-M-14_ and *bla*_CTX-M-15_ genes and ST405 *E. coli* carrying *bla*_CMY-2_ gene were predominant among the isolated *E. coli* strains. The majority of the ESBL/AmpC-producing isolates displayed diverse pulsed-field gel electrophoresis profiles. This study demonstrated that third-generation cephalosporin- and fluoroquinolone-resistant *E. coli* were widely distributed in companion animals. The detection of the pandemic ST131 clone carrying *bla*_CTX-M_-_14/15_ in companion animals presented a public health threat.

## 1. Introduction

*Escherichia coli* is a Gram-negative, rod-shaped facultative commensal bacterium commonly found in the gastrointestinal tract of humans and warm-blooded animals and rarely causes diseases. However, some strains have acquired genes that enable them to cause intestinal and extraintestinal infections. The most common strains associated with intestinal infections include enteropathogenic, enterotoxigenic, enteroinvasive, enteroaggregative, and enterohemorrhagic *E. coli* [1]. The disruption of normal intestinal anatomic barriers and subsequent spread of *E*. *coli* to adjacent tissue structures or the bloodstream could lead to extraintestinal infections such as urinary tract infections, meningitis, septicemia, and skin infections, especially in immunocompromised individuals [1,2,3].

Fluoroquinolones and third-generation cephalosporins are among the clinically important antimicrobials for the treatment of *E. coli* infection. However, the overuse or misuse of these antimicrobials in humans and companion animals has contributed to the emergence of cephalosporin- and fluoroquinolones-resistant strains. Alarmingly, the occurrence of *E. coli* co-resistant to these antimicrobials could lead to failure of treatment for serious infection in humans and companion animals [4,5].

Extended-spectrum β-lactamase (ESBL) and/or plasmid-mediated AmpC β-lactamase (pAmpC)-producing *E. coli* have been identified in companion animals worldwide [6,7]. In South Korea, we identified *bla*_CTX-M-14 -_carrying *E. coli* from a diseased dog for the first time in 2009 [8]. Since then, various ESBL and AmpC-producing *E. coli* have been isolated from companion animals [9,10]. The frequent and direct interactions between humans and companion animals may play a significant role in the dissemination of ESBL and AmpC-producing *E. coli* [11]. Therefore, this study aimed to determine the prevalence of third-generation cephalosporins and fluoroquinolone resistance in *E. coli* isolated from dogs and cats. Further investigations were also conducted to determine the molecular characteristics of ESBL and/or AmpC-producing *E. coli*.

## 2. Results

### 2.1. Prevalence of Cefovecin and Enrofloxacin Resistance

A total of 836 *E. coli* isolates were obtained from dogs (695) and cats (141) (Table 1). The overall cefovecin and enrofloxacin resistance rates were 17.1% and 21.2%, respectively. Cefovecin and enrofloxacin resistance rates in dog isolates (18.1% and 22.9%) were significantly higher than that of cat isolates (12.1% and 12.8%) (*p* < 0.05). *E. coli* isolated from the skin/ear and urine of dogs and cats exhibited relatively high resistance rates to cefovecin (24.2–25%) and enrofloxacin (23.4–34.1%). Cefovecin and enrofloxacin resistance varied with age groups. A relatively high resistance rate to these antimicrobials was found in isolates from dogs aged 11–15 years, followed by those from <1 year and 6–10 years age groups (Table 2). However, almost all of the cefovecin and enrofloxacin-resistant isolates from cats were identified from those aged <6 years. Generally, although the number of isolates from older animals (≥15 years) was fewer than those isolated from younger (≤5 years) age groups, cefovecin and enrofloxacin resistance rates were relatively high in isolates from adult animals (aged 11–15 years) compared with those from younger animals.

### 2.2. Distribution and Characterization of ESBL/AmpC-Producing E. coli

We selected 89 cefovecin and enrofloxacin-resistant isolates (70 from dogs and 11 from cats) for further assessment of ESBL/AmpC-production (Table 3). We identified five different types of *bla*_CTX-M_ types (*bla*_CTX-M-3_, *bla*_CTX-M-14_, *bla*_CTX-M-15_, *bla*_CTX-M-55_, and *bla*_CTX-M-65_) and two AmpC types (*bla*_CMY-2_ and *bla*_DHA_). *bla*_CTX-M-14_ and *bla*_CTX-M-15_-carrying isolates comprised the majority (73.2%, 41/56) of the *bla*_CTX-M_ types. Most of the *bla*_CTX-M_ -carrying isolates from dogs and all of the *bla*_CTX-M_-carrying isolates from cats were obtained from fecal samples. Indeed, only a very few *bla*_CTX-M-3_ and *bla*_CTX-M-55_ carrying isolates were found among isolates from urine samples of dogs. In addition, *bla*_CMY-2_ was detected in all AmpC-producing isolates, while *bla*_DHA_ was detected only in one isolate. Interestingly, the co-occurrence of *bla*_CTX-M_ and *bla*_CMY_ genes was noted in six dog isolates. 

### 2.3. Mechanisms of Quinolone Resistance

Sequencing analysis demonstrated that all the 89 cefovecin and enrofloxacin-resistant isolates possessed at least one mutation in the quinolone resistance-determining region (QRDR) (Table 4). The most frequently observed mutations in both dog and cat isolates were S83L and D87N in *gyrA* and S80I in *parC*. In addition, a total of 11 dog isolates carried the *qnr* genes (six *aac(6’)-Ib-cr*, four *qnrS*, and one *qnrB*), while only two cat isolates carried *qnrS*. Interestingly, *qnr* gene carriage did not cause a significant difference in fluoroquinolone MICs.

### 2.4. Molecular Characterization

**Figure 1:**
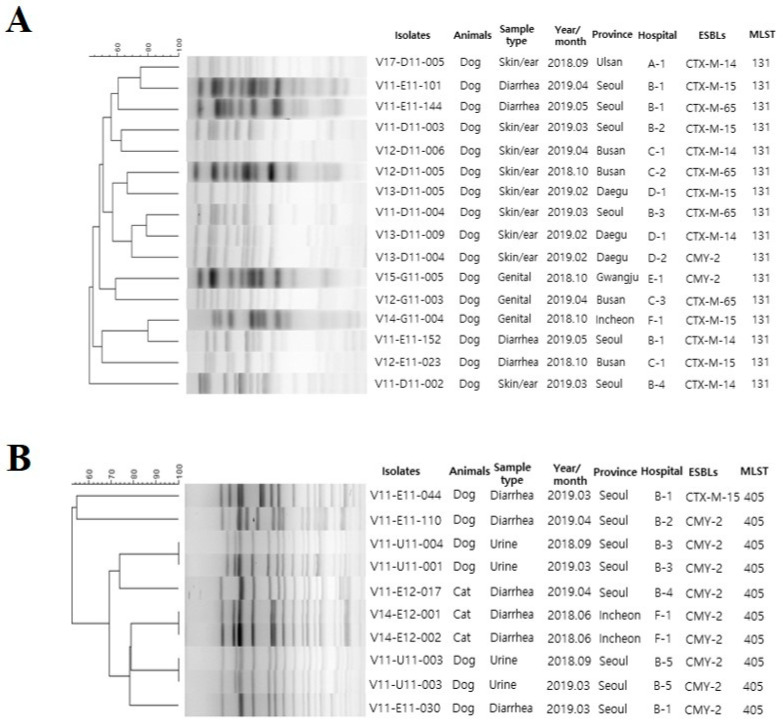
PFGE patterns *E. coli* ST131 (**A**) and *E. coli* ST405 (**B**) isolated from diseased dogs and cats in Korea.

Multi-locus sequence typing (MLST) revealed 25 STs, with 23 types in 78 dog isolates and 7 types in 11 cat isolates (Table 5). ST131 was the predominant ST in dog isolates followed by ST405, ST457, and ST38. ST131 *E. coli* was widely distributed in twelve hospitals that were located in six cities. The most predominant STs among cat isolates were ST405 and ST648. PFGE analysis revealed that all the ST131 *E. coli* isolates from dogs showed distinct PFGE profiles (40–80% similarity), indicating genetic variability among the isolates. Notably, four *bla*_CMY-2_-carrying ST405 *E. coli* from dogs and cats from different hospitals in Seoul and Incheon cities presented identical PFGE patterns (Figure 1).

## 3. Discussion

In this study, a significant proportion of *E. coli* isolated from diseased dogs and cats in Korea was identified as ESBL/AmpC-producers, and *bla*_CTX-M_- and/or *bla*_CMY-2_ -carrying isolates were further characterized using molecular techniques.

Third-generation cephalosporin-resistant isolates are regarded as a potential hazard to both animal and human health because they frequently exhibit multidrug resistance to clinically important antimicrobials [12]. In the present study, 18.1% and 12.1% of the *E. coli* isolated from dogs and cats were resistant to cefovecin. The cefovecin resistance rate in this study agreed with earlier studies from Hong Kong (20%) [13] and Australia (17%) [14]. However, it was lower than other reports in the UK (31%) [15] and Poland (28%) [16]. Interestingly, we identified a relatively high third-generation cephalosporin resistance in isolates from urine and skin/ear samples. This could be associated with the frequent use of these antimicrobials for the treatment of dermatitis and urinary tract infections in companion animals. A recent study has also shown the link between the use of third-generation cephalosporins and emergence of resistance to one or more of these antimicrobials in companion animals [17]. The high prevalence of fluoroquinolone-resistant *E. coli* among companion animals and humans is still a matter of concern [18].

In this study, the enrofloxacin resistance rate in dog isolates (23.2%) was almost twice the rate in cat (12.8%) isolates. This finding concurred with previous reports in France (19%) [19], South Africa (16%) [20], and the USA (16%) [21]. However, it was lower than those reported in China (61%) [22], Japan (40%) [23], and Greece (39%) [24]. The fluoroquinolone resistance is linked with mutations in topoisomerases or acquisition of plasmid-mediated quinolone resistance genes [25].

Resistance to multiple critically important antimicrobials poses significant public health concerns. In this study, about 11% of *E. coli* isolates were resistant to cefovecin and enrofloxacin. Resistance to both antimicrobials in *E. coli* isolated from companion animals has been reported in Korea [26] and other countries [27,28]. The frequent application of fluoroquinolones and third-generation cephalosporins in companion animals could lead to the emergence of *Enterobacteriaceae* resistant to both antimicrobials [29]. In addition, fluoroquinolone resistance in *E. coli* might trigger resistance to third-generation cephalosporins [5,28]. Previous studies showed that resistance to extended-spectrum cephalosporins and fluoroquinolones could also occur in other Gram-negative *Enterobacteriaceae*, such as *Klebsiella* spp. [30,31,32]. *Klebsiella pneumoniae* isolated from humans and companion animals can produce β-lactamase, making cephalosporin ineffective by hydrolyzing the β-lactam ring [10,33]. This extended-spectrum cephalosporin-resistant *Enterobacteriaceae* can directly spread and distribute between humans and companion animals [11].

It has been shown that age is an important factor in the development of bacterial infection and the emergence of antimicrobial resistance [34]. However, only limited reports are available on the prevalence of antimicrobial resistance in bacteria isolated from companion animals of different age groups. In agreement with Schwartz et al. [35], this study showed that cefovecin and enrofloxacin resistance rates were high in isolates identified from relatively old dogs (aged 11–15 years), providing evidence for the occurrence of antimicrobial resistance traits in isolates from relatively older age groups of animals. In contrast, some previously published data showed inconsistent resistance dynamics in isolates from animals of different age groups [36].

Diverse ESBL/AmpC-encoding genes have been detected in bacteria isolated from companion animals worldwide. In this study, the *bla*_CTX-M-14_ and *bla*_CTX-M-15_ genes were the most frequent ESBL genes in both dog and cat isolates. These genes were frequently reported in humans and companion animals in Korea [9]. In addition, *bla*_CMY-2_ was identified in 43.8% of *E. coli* isolated from dogs and cats. This gene was frequently detected in cephamycin-resistant *E. coli* recovered from humans and animals in many countries [31,37]. Notably, plasmids bearing the *bla*_CMY-2_ gene have also been reported in clinical and community isolates in humans in Korea [37]. These results emphasize the necessity of coordinated control of ESBL/AmpC-producing *E. coli* in humans and companion animals.

Mutations in *gyrA* and *parC* genes have been linked with high fluoroquinolone resistance [38]. In this study, we identified mutations in *gyrA* and *parC* genes in fluoroquinolone-resistant *E. coli* isolated from dogs and cats. The most common mutations were S83L and D87N in *gyrA* and S80I in *parC*. Hopkins et al. [39] also demonstrated that codons 83 and 87 of *gyrA* and codon 80 of *parC* were the most frequent mutation sites in *gyrA* and *parC.* In this study, about 14% of the enrofloxacin-resistant isolates harbor at least one PMQR gene, with *qnrB*, *qnrS*, and *aac(6′)-Ib-cr* being detected alone or in combination. Agreeing with this study, *E. coli* isolated from humans and companion animals have frequently been observed to carry *qnr* [28,40,41] and *aac(6’)-Ib-cr* [38,40,41,42,43] genes. In addition, all the enrofloxacin-resistant *E. coli* isolates exhibited high levels of resistance (MIC ≥ 16 mg/L), regardless of *qnr* gene carriage. Therefore, fluoroquinolone resistance in these isolates could be associated with the carriage of one or more PMQR-encoding genes as well as mutations in the QRDR [44]. Particularly, the co-occurrence of PMQR and *bla*_CTX-M_ and/or *bla*_CMY-2_ genes in isolates from companion animals constitutes a public health concern.

MLST is useful for assessing major changes of the lineages among isolates and understanding global epidemiology [45]. We observed 25 different *E. coli* STs, of which 23 were from dogs and 7 were from cats. Four of the STs (ST648, ST457, ST357, and ST405) were found in both dogs and cats. In this study, isolates belonging to diverse STs were recovered from most of the cities. This might be associated with the widespread distribution of some clones (ST131 and ST38) in Korea or due to the visits of animals to hospitals in different cities. Additionally, *E. coli* ST131 isolates were frequently detected in dogs from different animal hospitals in four cities. *E. coli* ST131 is a multidrug-resistant pandemic clone found in humans and companion animals [46,47]. This clone is responsible for various infections in humans [47,48]. Interestingly, this ST131 *E. coli* isolate was frequently reported to carry *bla*_CTX-M-15_ in many countries [49]. In Korea, *bla*_CTX-M-15_ carrying ST131 *E. coli* has been associated with a variety of invasive infections in humans [50]. The frequent contact between humans and companion animals necessities further studies on clonal relatedness between isolates from humans and companion animals. PFGE analysis showed that the *bla*_CTX-M_-carrying ST131 *E. coli* presented diverse patterns in dogs, which implies that most of the isolates acquired resistance individually. In contrast, a few *bla*_CMY-2_-carrying ST405 strains of dogs and cats exhibited similar PFGE patterns. This could be due to cross-contamination in the animal shelter or hospitals and/or staff or material movement within the same facility.

This study provides important insights into mechanisms of resistance and molecular profiles of cephalosporin and fluoroquinolone-resistant *E. coli* resistant strains isolated from companion animals. Since *E. coli* that carry resistance to clinically important antimicrobials can be transmitted between humans and companion animals, it is crucial to establish prevention and control strategies in veterinary practice settings. Furthermore, addressing concerns about the potential transmission of these infections to humans is of utmost importance.

## 4. Materials and Methods

### 4.1. Sample Collection

**Figure 2:**
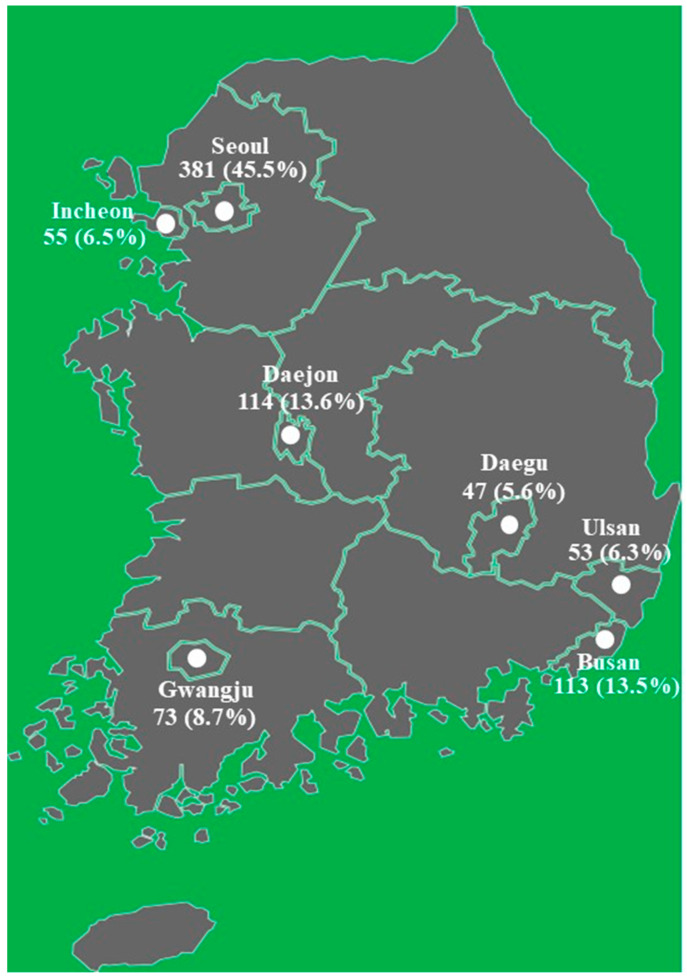
Location of *E. coli* isolates collection cities in Korea.

*E. coli* isolates were collected from seven different laboratories/centers participating in the Korean Veterinary Antimicrobial Resistance Monitoring System from 2018 to 2019. The isolates were recovered from the diarrhea, skin, ear canals, urine, and genitalia of dogs and cats. Samples were placed on ice and transported to the laboratories/centers within 6 h of collection. The number of isolates collected from different veterinary hospitals in each city is shown in Figure 2. However, the authors do not have information about the history of antimicrobial use in dogs and cats, and the number of samples considered for this study.

### 4.2. E. coli Isolation

Isolation and identification of *E*. *coli* were performed as described in our previous reports [51,52]. Briefly, swab samples were streaked on Eosin Methylene Blue (EMB) agar (Becton Dickinson, Sparks, NV, USA) and incubated at 37 °C for 24 h. Then, three suspected colonies were sub-cultured on MacConkey agar plates (MAC, BD, Spark, Baltimore, MD, USA) and incubated overnight at 37 ℃ for 24 h. Isolates were then confirmed by matrix-assisted laser desorption and ionization-time-of-flight mass spectrometry (MALDI-TOF, Biomerieux, Marcy L’Etoile, France). Only a single isolate per sample was considered for further assay [52].

### 4.3. Antimicrobial Susceptibility Testing

The antimicrobial susceptibility profiles of the isolates were determined by the broth microdilution methods based on the Clinical and Laboratory Standards Institute (CLSI) guidelines [53], using commercially available Sensititre plates COMPGN1F (Thermo Trek Diagnostics, Waltham, MA, USA). The isolates were tested for susceptibility toward ciprofloxacin, enrofloxacin, marbofloxacin, nalidixic acid, and ofloxacin. *E. coli* ATCC25922 was used as a quality reference strain. The results were interpreted according to the CLSI guidelines [53].

### 4.4. Mechanisms of Antimicrobial Resistance

A polymerase chain reaction (PCR) assay was performed to detect the presence of *bla*_CTX-M_ and AmpC genes using the previously described primers (Supplementary Material Table S1) [12]. Sequence analysis was performed using ABI3730XL DNA sequence analyzer (SolGent, Daejeon, Republic of Korea) and comparison with known sequences was performed with the Basic Local Alignment Search Tool (BLAST) programs at the National Center for Biotechnology Information website (www.ncbi.nim.nih.gov/BLAST, accessed on 10 August 2022). Multiplex PCR was used to detect plasmid-mediated quinolone resistance (PMQR) genes such as *qnrB*, *qnrS*, and *aac(6’)-Ib-cr* using conditions and primers described previously (Table S1) [12]. In addition, the quinolone resistance-determining regions (QRDR) of *gyrA* and *parC* were amplified using primers described previously (Table S1) [54]. Following that, the purified PCR products were sequenced by an automated ABI prism 3700 analyzer (Applied Biosystems, Foster City, CA, USA) and compared to those of standard *E. coli* K-12 strain using the BLAST and exPASY proteomics tools (www.expasy.ch/tools/similarity, accessed on 10 August 2022).

### 4.5. Molecular Characterization

Multi-locus sequencing typing (MLST) was carried out to investigate the clonal relationship of the cefovecin- and enrofloxacin-resistant isolates according to the previously described method [55]. The following six housekeeping genes were amplified and sequenced using specific primers: *adk*, *fumC*, *gyrB*, *icd*, *mdh*, *purA*, and *recA*. Allelic profile and sequence types (STs) were determined using web-based MLST databases for *E. coli* (https://pubmlst.org/databases/, accessed on 12 September 2022). In addition, the genetic diversity of the ESBL/AmpC-producing isolates was assessed by pulsed-field gel electrophoresis (PFGE) of chromosomal DNA digested with *Xba*I (Takara Inc., Shiga, Japan) [56]. The relatedness of the isolates was then determined using the unweighted pair group approach with the arithmetic average algorithm (UPGMA) based on the Dice similarity index (Bionumerics software, version 4.0; Applied Maths, Sint-Martens-Latem, Belgium).

### 4.6. Statistical Analysis

The statistical analysis was performed using the SPSS software (version 25, Armonk, NY, USA), which included the independent samples *t*-test, Fisher’s exact test, and chi-square test. *p*-Values < 0.05 were considered statistically significant.

## 5. Conclusions and Future Perspectives

The current study demonstrated that *E. coli* isolated from companion animals (dogs and cats) exhibited resistance to cefovecin and enrofloxacin. ESBL/AmpC-producing *E. coli* carried predominantly *bla*_CTX-M-14_, *bla*_CTX-M-15_, and *bla*_CMY-2_ genes. Fluoroquinolone-resistant isolates frequently possessed S83L and D87N mutations in QRDR of *gyrA* and *parC* as well as carry PMQR genes (*aac(6’)-Ib-cr*, *qnrS*, and *qnrB*). Particularly, *bla*_CTX-M-15_-carrying pandemic *E. coli* ST131 strain was most frequent among *E. coli* isolated from dogs. The study limitations include the absence of data on the number of samples by sex and season and the severity of the animals’ ailment. However, this study provides useful insight into third-generation cephalosporin and fluoroquinolones resistance in companion animals. Therefore, long-term surveillance of resistance patterns and judicious selection of antimicrobials is required to prevent the spread and evolution of antibiotic-resistant *E. coli* among humans and companion animals.

## Figures and Tables

**Table 1:** Cefovecin and enrofloxacin resistance in *E. coli* isolated from diseased dogs and cats during 2018–2019.

Animals	Samples	No. of *E. coli* Isolates	Resistance % (No. of Isolates)			*p*-Value
			Cefovecin	Enrofloxacin	Cefovecin and Enrofloxacin	
Dogs	Diarrhea	446	17.9 (80) ^a,A^	22.2 (99) ^a,A^	11.2 (50) ^a,A^	0.192
	Skin/ear	91	24.2 (22) ^b,A^	34.1 (31) ^b,A^	18.7 (17) ^b,A^	0.210
	Urine	64	25.0 (16) ^b,A^	23.4 (15) ^a,A^	10.9 (7) ^a,A^	0.403
	Genital	94	8.5 (8) ^c,A^	14.9 (14) ^c,A^	5.3 (5) ^c,A^	0.421
	Subtotal	695	18.1 (126) ^A^	22.9 (159) ^A^	11.4 (79) ^A^	0.542
Cats	Diarrhea	128	12.5 (16) ^a,B^	14.1 (18) ^a,B^	8.6 (11) ^a,A^	0.999
	Skin/ear	4	25.0 (1) ^b,A^	0 (0) ^b,B^	0 (0) ^b,B^	–
	Urine	4	0 (0) ^c,B^	0 (0) ^b,B^	0 (0) ^b,B^	–
	Genital	5	0 (0) ^c,B^	0 (0) ^b,B^	0 (0) ^b,B^	–
	Subtotal	141	12.1 (17) ^B^	12.8 (18) ^B^	7.8 (11) ^A^	0.829
Total		836	17.1 (143)	21.2 (177)	10.8 (90)	0.562

The values with different lowercase letters as superscript (a, b, or c) represent significant statistical differences among sample groups in the same species in the same column. The different uppercase letters as superscript (A or B) indicates a significant difference between species within the same column (*p* < 0.05). *p*-value denotes the statistical significance between cefovecin-resistant and enrofloxacin-resistant isolates.

**Table 2:** Distribution of cefovecin- and enrofloxacin-resistant *E. coli* among different age groups of diseased dogs and cats.

Animals	Ages (Year)	Resistance % (No. of Isolates)		
		Cefovecin	Enrofloxacin	Cefovecin and Enrofloxacin
Dogs	<1 (n = 69)	17.4 (12) ^a,A^	26.1 (18) ^a,A^	14.5 (10) ^a,A^
	1–5 (n = 242)	14.0 (34) ^a,A^	14.0 (34) ^b,A^	6.2 (15) ^b,A^
	6–10 (n = 164)	16.5 (27) ^a,A^	22.6 (37) ^a,A^	11.6 (19) ^a,A^
	11–15 (n = 130)	28.5 (37) ^b,A^	43.1 (56) ^c,A^	23.1 (30) ^c,A^
	>15 (n = 22)	9.1 (2) ^c,A^	13.6 (3) ^b,A^	4.5 (1) ^b,A^
	Unknown (n = 68)	20.6 (14)	19.1 (13)	5.9 (4)
	Subtotal (n = 695)	18.1 (126) ^A^	23.2 (161) ^A^	11.4 (79) ^A^
Cats	<1 (n = 51)	7.8 (4) ^a,B^	7.8 (4) ^a,B^	2.0 (1) ^a,B^
	1–5 (n = 61)	14.8 (9) ^b,A^	16.4 (10) ^b,A^	9.8 (6) ^b,A^
	6–10 (n = 8)	0 (0) ^c,B^	0 (0) ^c,B^	0 (0) ^c,B^
	11–15 (n = 2)	50.0 (1) ^d,B^	50.0 (1) ^d,B^	50.0 (1) ^d,B^
	>15 (n = 1)	0 (0) ^c,B^	0 (0) ^c,B^	0 (0) ^c,B^
	Unknown (n = 18)	16.7 (3)	16.7 (3)	16.7 (3)
	Subtotal (n = 141)	12.1 (17) ^B^	12.8 (18) ^B^	7.8 (11) ^A^
Total (n = 836)		17.1 (143)	21.4 (179)	10.8 (90)

The values with different lowercase letters as superscript (a, b, c, or d) represent significant differences among the groups in the same species in the same column and the different uppercase letters as superscript (A or B) indicate significant differences between different species within the same column (*p* < 0.05).

**Table 3:** Distribution of ESBL/AmpC-producing *E. coli* isolated from diseased dogs and cats.

ESBL/AmpC Gene	% (No.) of Isolates						
	Dogs					Cats	Total (n = 89)
	Diarrhea (n = 50)	Skin/Ear (n = 16)	Urine (n = 7)	Genital (n = 5)	Subtotal (n = 78)	Diarrhea (n = 11)	
*bla*_CTX-M-3_ + *bla*_CMY-2_	0 (0)	0 (0)	14.3 (1)	0 (0)	1.3 (1)	0 (0)	1.1 (1)
*bla*_CTX-M-15_	20.0 (10)	31.3 (5)	0 (0)	20.0 (1)	20.5 (16)	9.1 (1)	19.1 (17)
*bla*_CTX-M-15_ + *bla*_CMY-2_	4.0 (2)	0 (0)	0 (0)	0 (0)	2.6 (2)	0 (0)	2.2 (2)
*bla*_CTX-M-55_	4.0 (2)	0 (0)	14.3 (1)	0 (0)	3.8 (3)	0 (0)	3.4 (3)
*bla*_CTX-M-55_ + *bla*_CMY-2_	2.0 (1)	0 (0)	14.3 (1)	0 (0)	2.6 (2)	0 (0)	2.2 (2)
*bla*_CTX-M-14_	20.0 (10)	43.8 (7)	0 (0)	0 (0)	21.8 (17)	36.4 (4)	23.6 (21)
*bla*_CTX-M-14_ + *bla*_CMY-2_	2.0 (1)	0 (0)	0 (0)	0 (0)	1.3 (1)	0 (0)	1.1 (1)
*bla*_CTX-M-65_	10.0 (5)	2.0 (2)	0 (0)	20.0 (1)	10.3 (8)	9.1 (1)	10.1 (9)
*bla*_CMY-2_	38.0 (19)	6.3 (1)	57.1 (4)	60.0 (3)	34.6 (27)	45.5 (5)	36.0 (32)
*bla*_CMY-2_ + *bla*_DHA_	0 (0)	6.3 (1)	0 (0)	0 (0)	1.3 (1)	0 (0)	1.1 (1)

**Table 4:** Mechanisms of quinolone resistance of *E. coli* isolates from dogs and cats.

Point Mutations within the QRDR		PMQR	No. of Isolates	Minimum Inhibition Concentration_50_ (MIC_50_) (mg/L)				
*gyrA*	*parC*			ENR	MAR	CIP	NAL	OFL
Dog isolates (n = 78)								
S83L	S80I	*qnrS* + *aac(6’)-Ib-cr*	1	32	16	32	256	16
S83L, D87G	E84K	–	1	32	16	16	256	16
S83L, D87Y	S80I	–	18	64	32	32	256	32
S83L, D87N	S80I	–	34	64	32	32	256	32
		*qnrB*	1	32	16	16	256	16
		*qnrS*	3	32	16	8	256	32
		*aac(6’)-Ib-cr*	2	64	16	32	256	32
S83L, D87N	S80I, E84G	–	2	32	16	16	256	16
	S80I, E84V	–	13	64	32	32	256	32
		*aac(6’)-Ib-cr*	3	64	16	64	256	16
Cat isolates (n = 11)								
S83L	S80I	*qnrS*	1	64	32	32	256	32
S83L, D87N	S80I	–	7	64	16	32	256	32
		*qnrS*	1	64	32	32	256	32
S83L, D87Y	S80I	–	2	32	16	16	256	16

CIP, ciprofloxacin; ENR, enrofloxacin; MAR, marbofloxacin; NAL, nalidixic acid; OFL, ofloxacin. MIC_50_, the concentration at which 50% of the isolates were inhibited.

**Table 5:** MLST profiles ESBL/AmpC-producing *E. coli* isolated from diseased dogs and cats in Korea.

ST Type	No. of Isolates		Province	Hospital	Resistance Gene
	Dog	Cat			
131	16	0	Seoul (n = 6), Busan (n = 4), Daegu (n = 3), Incheon (n = 1), Ulsan (n = 1), Gwangju (n = 1)	H-2 (n = 1), H-4 (n = 2), H-5 (n = 1), H-7 (n = 2), H-10 (n = 1), H-11 (n = 1), H-18 (n = 1), H-24 (n = 1), H-25 (n = 3), H-26 (n = 1), H-28 (n = 1), H-30 (n = 1)	*bla*_CTX-M-14_ (n = 5), *bla*_CTX-M-15_ (n = 5), *bla*_CTX-M-65_ (n = 4), *bla*_CMY-2_ (n = 2)
405	8	3	Seoul (n = 8), Incheon (n = 2), Daegu (n = 1)	H-4 (n = 1), H-12 (n = 2), H-13 (n = 2), H-22 (n = 2), H-33 (n = 2), H-15 (n = 1), H-25 (n = 1)	*bla*_CMY-2_ (n = 9), *bla*_CTX-M-3_ + *bla*_CMY-2_ (n = 1), *bla*_CTX-M-15_ (n = 1)
457	8	1	Seoul (n = 5), Incheon (n = 3), Gwangju (n = 1)	H-30 (n = 2), H-13 (n = 1), H-17 (n = 1), H-18 (n = 1), H-19 (n = 1), H-27 (n = 1), H-32 (n = 1), Unknown (n = 1)	*bla*_CMY-2_ (n = 5), *bla*_CTX-M-15_ (n = 3), *bla*_CTX-M-15_ + *bla*_CMY-2_ (n = 1)
38	7	1	Seoul (n = 3), Ulsan (n = 2), Daejeon (n = 1), Busan (n = 1), Gwangju (n = 1)	H-1 (n = 1), H-6 (n = 1), H-7 (n = 1), H-22 (n = 1), H-25 (n = 2), H-28 (n = 1), H-29 (n = 1)	*bla*_CTX-M-14_ (n = 6), *bla*_CTX-M-15_ (n = 2)
648	5	3	Seoul (n = 7), Busan (n = 1)	H-13 (n = 2), H-11 (n = 1), H-16 (n = 1), H-20 (n = 1), H-23 (n = 1), H-24 (n = 1), H-25 (n = 1)	*bla*_CTX-M-14_ (n = 4), *bla*_CMY-2_ (n = 3), *bla*_CTX-M-15_ (n = 1)
155	5	0	Seoul (n = 4), Gwangju (n = 1)	H-2 (n = 1), H-12 (n = 1), H-16 (n = 1), H-20 (n = 1), H-34 (n = 1)	*bla*_CMY-2_ (n = 3), *bla*_CTX-M-14_ (n = 1), *bla*_CTX-M-15_ (n = 1)
2003	5	0	Seoul (n = 2), Daegu (n = 1), Busan (n = 1), Gwangju (n = 1)	H-3 (n = 1), H-8 (n = 1), H-14 (n = 1), H-25 (n = 1), Unknown (n = 1)	*bla*_CMY-2_ (n = 2), *bla*_CTX-M-14_ (n = 1), *bla*_CTX-M-14_ + *bla*_CMY-2_ (n = 1), *bla*_CTX-M-55_ (n = 1)
410	4	0	Seoul (n = 4)	H-21 (n = 2), H-19 (n = 1), H-20 (n = 1)	*bla*_CMY-2_ (n = 4)
224	3	0	Seoul (n = 2), Gwangju (n = 1)	H-25 (n = 2), H-1 (n = 1)	*bla*_CMY-2_ (n = 2), *bla*_CTX-M-65_ (n = 1)
1193	3	0	Seoul (n = 1), Daejeon (n = 1), Ulsan (n = 1)	H-6 (n = 1), H-13 (n = 1), H-28 (n = 1)	*bla*_CTX-M-55_ (n = 2), *bla*_CTX-M-14_ (n = 1)
354	1	1	Seoul (n = 1), Busan (n = 1)	H-9 (n = 1), H-23 (n = 1)	*bla*_CTX-M-15_ (n = 1), *bla*_CMY-2_ (n = 1)
744	2	0	Incheon (n = 2)	H-31 (n = 1), H-32 (n = 1)	*bla*_CTX-M-65_ (n = 2)
34	0	1	Seoul (n = 1)	H-17 (n = 1)	*bla*_CTX-M 14_ (n = 1)
69	1	0	Seoul (n = 1)	H-13 (n = 1)	*bla*_CTX-M 14_ (n = 1)
105	1	0	Seoul (n = 1)	H-23 (n = 1)	*bla*_CTX-M-15_ + *bla*_CMY-2_ (n = 1)
162	1	0	Seoul (n = 1)	H-25 (n = 1)	*bla*_CTX-M-65_ (n = 1)
372	1	0	Incheon (n = 1)	H-33 (n = 1)	*bla*_CTX-M-15_ (n = 1)
450	1	0	Seoul (n = 1)	H-22 (n = 1)	*bla*_CTX-M-55_ + *bla*_CMY-2_ (n = 1)
1011	1	0	Seoul (n = 1)	H-15 (n = 1)	*bla*_CMY-2_ (n = 1)
1196	1	0	Seoul (n = 1)	H-12 (n = 1)	*bla*_CMY-2_ (n = 1)
2159	0	1	Ulsan(n = 1)	H-28 (n = 1)	*bla*_CTX-M-65_ (n = 1)
2245	1	0	Seoul (n = 1)	H-15 (n = 1)	*bla*_CTX-M-55_ + *bla*_CMY-2_ (n = 1)
4516	1	0	Seoul (n = 1)	H-13 (n = 1)	*bla*_CTX-M-14_ (n = 1)
5150	1	0	Gwangju (n = 1)	H-1 (n = 1)	*bla*_CTM-M-15_ (n = 1)
5869	1	0	Gwangju (n = 1)	H-1 (n = 1)	*bla*_CTX-M-15_ (n = 1)

## Data Availability

All data generated for this study are contained within the article.

## Supplementary Materials

The following supplementary materials are available at https://www.mdpi.com/article/10.3390/antibiotics12040745/s1, Table S1: Lists of primer sequences and PCR conditions. References [57,58,59,60,61,62,63] are cited in the supplementary materials.
