# Supplementary material for: Prevalence and Characterization of Extended-Spectrum β-Lactamase-Producing Escherichia coli Isolated from Dogs and Cats in South Korea
Source: Antibiotics (Basel). 2023 Apr 13;12(4):745. doi: 10.3390/antibiotics12040745 (PMC10135382; doi:10.3390/antibiotics12040745)
Supplement: Supplementary file 1 [file antibiotics-12-00745-s001.zip › antibiotics-2315702-supplementary.pdf]

Table S1. List of primers sequences and PCR conditions

Primer	Sequence (5'-3')	Size (bp)	PCR condition	References
CTX-M-Universal[a]	ATGTGCAGYACCAGTAARGTKATGGC<br>TGGGTRAARTARGTSACCAGAAYCAGCGG	593	35 cycles; 95°C 1min + 60°C 1min + 72°C 1min	[1]
CTX-M-1 families[a]	AAGACTGGGTGTGGCATTGA<br>AGGCTGGGTGAAGTAAGTGA	670	35 cycles; 95°C 1min + 60°C 1min + 72°C 1min	[1]
CTX-M-2 families[a]	CTGGAAGCCCTGGAGAAAAG<br>TACCTCGCTCCATTTATTGC	789	35 cycles; 95°C 1min + 60°C 1min + 72°C 1min	[1]
CTX-M-8 families[a]	GCCTGTATTTCGCTGTTG<br>TGTCATTCGTCGTACCATAA	686	35 cycles; 95°C 1min + 60°C 1min + 72°C 1min	[1]
CTX-M-9 families[a]	GCTTTATGCGCAGACGAGTG<br>GCCAGATCACCGCAATATCA	703	35 cycles; 95°C 1min + 60°C 1min + 72°C 1min	[1]
CTX-M-1-full[a]	CAGCGCTTTTGCCGTCTAAG<br>AAAAATGATTGAAAGGTGGT	1100	35 cycles; 94°C 30sec + 55°C 30 sec + 72°C 1min	[2]
CTX-M-9-full[a]	GAAGCAGTCTAAATTCTTCGTGAAATAG<br>GGGCCAGTTGGTGATTTGA	1100	35 cycles; 94°C 30sec + 60°C 30 sec + 72°C 1min	[2]
DHA[b]	AACTTTCACAGGTGTGCTGGGT<br>CCGTACGCATACTGGCTTTGC	405	25 cycles; 94°C 30 sec + 64°C 30 sec + 72°C 1min	[3]
QnrB[c]	GATCGTGAAAGCCAGAAAGG<br>ATGAGCAACGATGCCTGGTA	476	35 cycles; 94°C 1min + 58°C 1min + 72°C 1min	[4]
QnrS[c]	ACTGCAAGTTCATTGAACAG<br>GATCTAAACCGTCGAGTTCG	431	35 cycles; 94°C 1min + 56°C 1min + 72°C 1min	[5]
acc (6′)-Ib-cr[c]	TGACCTTGCGATGCTCTATG<br>TTAGGCATCACTGCGTGTTC	508	35 cycles; 94°C 30sec + 60°C 30sec + 72°C 30sec	[6]
gyrA[d]	ACGTACTAGGCAATGACTGG<br>AGAAGTCGCCGTCGATAGAAC	610	30 cycles; 94ºC 1 min + 55ºC 1 min + 72ºC 1 min	[7]
parC[d]	TGTATGCGATGTCTGAACTG<br>CTCAATAGCAGCTCGGAATA	950	30 cycles; 94ºC 1 min + 55ºC 1 min + 72ºC 1 min	[7]

[a] Primer sequences used in bla_{CTX-M}

[b] Primer sequences used in bla_{CMY}

[c] Primer sequences used in plasmid mediated quinolone resistance (PMQR) genes

[d] Primer sequences used in determination of quinolone resistance determining region (QRDR)

References

1. Batchelor, M.; Threlfall, E.J.; Liebana, E. Cephalosporin Resistance among Animal-Associated Enterobacteria: A Current Perspective. *Expert Rev. Anti. Infect. Ther.* **2005**, *3*, 403–417.
2. Tamang, M.D.; Nam, H.-M.; Gurung, M.; Jang, G.-C.; Kim, S.-R.; Jung, S.-C.; Park, Y.H.; Lim, S.-K. Molecular Characterization of CTX-M β-Lactamase and Associated Addiction Systems in *Escherichia coli* Circulating among Cattle, Farm Workers, and the Farm Environment. *Appl. Environ. Microbiol.* **2013**, *79*, 3898–3905.
3. Pérez-Pérez, F.J.; Hanson, N.D. Detection of Plasmid-Mediated AmpC β-Lactamase Genes in Clinical Isolates by Using Multiplex PCR. *J. Clin. Microbiol.* **2002**, *40*, 2153–2162.
4. Dorji, T.M.; Yong, S.S.; Jae-Young, O.; Young, K.H.; Chul, L.J.; Chul, L.Y.; Taek, C.D.; Jungmin, K. Plasmid-Mediated Quinolone Resistance Determinants QnrA, QnrB, and QnrS among Clinical Isolates of *Enterobacteriaceae* in a Korean Hospital. *Antimicrob. Agents Chemother.* **2008**, *52*, 4159–4162, doi:10.1128/AAC.01633-07.
5. A., J.G. AmpC β-Lactamases. *Clin. Microbiol. Rev.* **2009**, *22*, 161–182, doi:10.1128/CMR.00036-08.
6. Kim, J.; Bae, I.K.; Jeong, S.H.; Chang, C.L.; Lee, C.H.; Lee, K. Characterization of IncF Plasmids Carrying the Bla CTX-M-14 Gene in Clinical Isolates of *Escherichia coli* from Korea. *J. Antimicrob. Chemother.* **2011**, *66*, 1263–1268.
7. Everett, M.J.; Jin, Y.F.; Ricci, V.; Piddock, L.J. Contributions of Individual Mechanisms to Fluoroquinolone Resistance in 36 *Escherichia coli* Strains Isolated from Humans and Animals. *Antimicrob. Agents Chemother.* **1996**, *40*, 2380–2386.
